# Naturalistic sleep tracking in a longitudinal cohort: Uncertainty and bias in short duration sampling

**DOI:** 10.1371/journal.pone.0334950

**Published:** 2025-11-03

**Authors:** Balaji Goparaju, Glen de Palma, Matt T. Bianchi

**Affiliations:** Apple Inc., Cupertino, California, United States of America; Portugal Football School, Portuguese Football Federation, PORTUGAL

## Abstract

**Background:**

Despite broad interest in the health implications of sleep duration, traditional measurements via polysomnography or actigraphy are often limited to one or a few nights per person. Inferential uncertainty remains an important issue for interpreting descriptive statistics in this common research setting.

**Methods:**

This retrospective analysis of observational data used a combined approach of simulated data and real-world data (30–365 nights) analysis from over 35,000 participants who provided informed consent to participate in the Apple Heart and Movement Study and elected to contribute sleep data.

**Results:**

Simulations demonstrate that the degree of uncertainty and bias, compared to truth defined by 1000 simulated nights, depended on several factors: sub-sample size, the simulated distribution (normal versus skewed), and the computed metrics of central tendency (mean, median) and dispersion (standard deviation (SD), interquartile range (IQR)). For example, the SD computed from n = 7 observations from a simulated normal distribution (7 ± 1 hours) showed a median 6.7% under-estimation bias, and an uncertainty range with IQR from 24% under- to 14.7% over-estimation. Defining ground truth with a small sample (7–14 nights) yielded overly optimistic estimates of bias and uncertainty when sub-sampled. Real-world sleep duration data, when randomly sub-sampled and compared to longer observations within-participant, showed similar SD bias and rates of convergence as the normal distribution simulations. Sub-sampled sleep stage durations also varied substantially from “true” values computed from longer observations. Finally, simulated cohorts with sleep durations of 7 ± 1 hours mixed with a subset of 6 ± 1 hours sleepers showed that a random single-night observation of “short sleep” (6 hours) is more likely from random variation of a 7-hour sleeper, than from an actual 6-hour sleeper. Extending the mean duration calculation to n = 7 nights mitigates this mis-classification risk.

**Conclusion:**

The simulation and empiric data approaches both suggest that bias and uncertainty due to sub-sampling depend on: a) the sample size of observations within each participant, b) the descriptive statistic used to capture centrality or dispersion, and c) the distribution shape of the data (normal or skewed). Longer duration tracking provides important and tangible benefits to reduce bias and uncertainty in sleep health research that historically relies on small observation windows.

## 1. Introduction

Although sleep is widely understood as a pillar of health and wellbeing, the objective measurement of sleep in research studies has been rather limited to short duration tracking via either gold-standard laboratory polysomnography (PSG) or approximations of sleep via actigraphy in the real-world. For example, the epidemiology of sleep stage architecture, described in two meta-analyses, is derived mainly from 1–3 nights of PSG data [1,2]. Similarly, in a recent review focused on nightly variability of sleep, actigraphy studies were mostly 7–14 nights in duration, with only seven of the 53 studies using actigraphy for more than 14 nights (and of those, the largest sample size was 141 subjects) [3]. Given the nightly variability in sleep duration, in part due to multiple biological, sociological, and behavioral influences on sleep [4], capturing 1–14 nights may be an under-sampled view of a person’s sleep patterns.

Even the use of self-reported diary entries, which in principle could continue for extended periods, are often used for relatively short time windows into sleep patterns. For example, the insomnia clinical guideline recommends a “two-week sleep log to identify sleep-wake times, general patterns, and day-to-day variability” [5]. The consensus sleep diary, which was designed primarily for insomnia research but is general in its content, is a one-week template but does not indicate how many weeks ought to be tracked [6]. The health and wellbeing implications of both self-reported and objective sleep tracking may be limited by short duration observation windows, with little data-driven evidence to rationalize any particular duration. Quantifying some of the statistical uncertainties that might accompany relatively short duration tracking could improve a range of research efforts in this space. Although attempts have been made to address various aspects of reliability of objective or self-reported sleep, most have used only 7 or 14 nights to define the “truth” against which sub-sampling was compared [7–10].

We used a combination of two approaches: 1) simulations with known distributions, and 2) real-world objective longitudinal data from participants who consented to participate in the Apple Heart and Movement Study [11,12]. We hypothesized that sub-sampling a range of nights from individuals with long-term objective sleep observations (from which “true” values can be computed) would help quantify uncertainty and bias when measures of centrality and dispersion are computed from short duration samples. Sub-sampling from simulated distributions provides bounding context under simplifying assumptions, i.e., uncertainty and/or bias of computed metrics attributable solely to sub-sampling. These results set the stage to then interpret sub-sampling results from empiric sleep observations from longitudinal real world tracking, where neither the true distribution nor its stationarity can be assumed. For comparison with a different longitudinal health tracking metric, we also performed sub-sampling of objective exercise data from the same study cohort. The results provide statistical inference context when interpreting small observation windows of any health metric that varies over time. The simulated and empiric data analysis suggests that uncertainty and bias from short durations is influenced by the underlying data distribution and the summary metric of interest (e.g., measures of central tendency vs measures of dispersion). From a pragmatic perspective, sub-sampling across a range of duration provides estimates of convergence rates, such that hypothesis-specific goals can be considered in terms of trade-offs of participant burden and how close to the theoretical truth a given observation window might be.

## 2. Methods

### 2.1. AHMS overview

This study included participants who consented to participate in the Apple Heart and Movement Study (AHMS) [12], and opted in to sharing HealthKit data streams including sleep tracking and physical activity data. This population includes adults residing in the United States who own an iPhone and an Apple Watch. This study collects a variety of data types including objective sleep and exercise data via HealthKit. Participants opt in to sharing data, and can withdraw from the study at any time. The current analysis represents retrospective analsysis of observational data from this cohort.

### 2.2. Simulations

Simulations were performed in Microsoft Excel and plotted with GraphPad Prism. Normal distribution data was generated in Excel, using the function NORM.INV(RAND(),mean, standard deviation (SD), and skewed distributions were generated in Excel using the functions BETA.INV(RAND(), alpha, beta) or LOGNORM.INV(RAND(), mean, SD). For example, in the normal distribution simulation for total sleep time (TST), a mean of 7 hours and standard deviation (SD) of 1 hour would be similar to self-reported variability in adults [13] and similar to that seen in the AHMS cohort.

### 2.3. Study population

AHMS was approved by the Advarra Central Institutional Review Board, and registered to ClinicalTrials.gov (ClinicalTrials.gov Identifier: NCT04198194). Informed consent is provided electronically within the Apple Research app. Participation does not require sleep tracking. However a subset of participants do track and opt in to sharing sleep data. In the current work, we restricted sleep analysis to when the data source was the first-party sleep application on the Apple Watch. To be included in the current analysis, we pre-defined the minimum data requirements and time window. For the sleep and exercise analysis, we required at least 90 days with sleep duration of at least 4 hours, in a window between Sept 1, 2021, and September 1, 2022 (n = 40,441). Sleep duration values <4 or >13 were excluded, and exercise values <3 or >300 were excluded. For the sleep stages analysis, we required at least 30 days with sleep duration of at least 4 hours in a window between October 15, 2022, and April 15, 2023 (n = 36,956 participants), to correspond to the availability of first party Apple Watch sleep staging. In both cases, only sleep data from first party Apple Watch source was included. There were no specific power calculations employed with respect to arriving at this analysis set, and no other inclusion or exclusion criteria applied.

### 2.4. Data types

Sleep data was obtained from HealthKit, and the total sleep duration was computed for each night, using only Apple Watch first party sleep tracking data. During the time period analyzed for the sleep and exercise data (2021–2022), Apple Watch sleep tracking provided a binary output, asleep or not, for those onboarded to the sleep experience and tracking sleep. As of iOS 16 and WatchOS 9, the sleep feature was updated to include sleep stage information, for a total of four states (awake, Core Sleep, Deep Sleep, Rapid eye movement (REM) sleep) [14].

Exercise data was obtained from HealthKit, as the number of minutes of exercise, which is captured in one of the three activity rings on the Apple Watch. Capturing exercise minutes happens passively during routine watch wear. User-indicated workouts entered using the Workout app also contribute to exercise minutes, but we made no distinction in the current analysis whether exercise minutes came from a logged workout or not; we used the total minutes of exercise recorded in HealthKit per day. This daily exercise value is the sum of exercise minutes in a 24-hour period from midnight to midnight.

The retrospective analysis was performed on data that was accessed on 07/31/2023 for this investigation. The authors did not have access to information that could identify individual participants during or after data collection.

### 2.5. Analysis of real-world health data

Statistical analyses and generation of plots were done in Python [15–17] for sleep and exercise data from AHMS participants. Where p-values are incorporated (for tests of normality and log-normality using the Shapiro-Wilk test), we used a 0.05 threshold for significance. When sub-sampling was performed in simulated data, the values are independent by definition, such that sub-sampled points can be considered part of a stationary process. By contrast, empiric sleep and exercise data from AHMS participants cannot be assumed independent or stationary. Real world data was thus sampled at random, within each participant. As such, the temporal order of sleep and exercise data was not preserved or aligned by date, but each participants data remains intact. For example, when taking a random sub-sample of 7 nights, each individual would have 7 of their nights chosen, without any mixing of data across individuals. As such, certain non-stationarities would not be captured such as weekend effects or other transient changes (e.g., illness, vacation) that could appear if sequential data with preserved dates and alignment across participants was used. For example, weekend related effects can be seen in sequential sub-sampling, whether repeated or in isolation (S1 Fig in S1 File). We also performed additional analysis of consecutive nights of empiric sleep data for comparison purposes. We did not perform adjustments for demographics or health status. We did not perform imputation for days or nights with missing data.

## 3. Results

### 3.1. Simulations

**Fig 1:**
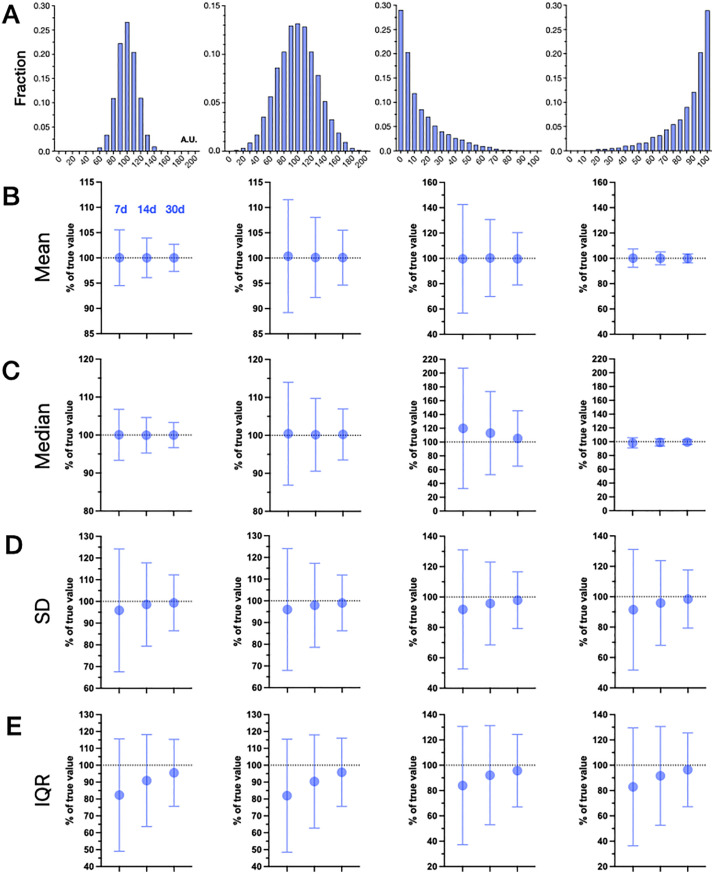
Descriptive statistics computed from sub-samples drawn from two normal and two skewed simulated distributions. **A.** Frequency histograms from four simulated distributions: two normal distributions with same mean (100 a.u.) but different SD values (15 vs 30 a.u.), and two skewed beta distributions with right vs left tails (right tail with alpha 0.5, beta 3.0, and left tail with alpha 3.0 and beta 0.5), scaled to be in the range of 0-100 a.u. The plots are derived from 5000 samples in each case, with bin size of 10 or 5 for normal vs skewed distributions, respectively. The next four rows show computed measures of centrality (mean (**B**) and median **C)**) and dispersion (SD (**D**) and IQR **(E)**), computed from sub-samples drawn from the corresponding distributions shown in row **A.** In each panel, random draws of size n = 7, n = 14, or n = 30, are summarized as the mean (circles) and standard deviation (error bars; SD) computed from of n = 5000 simulated subjects. The summary metrics are normalized and reported as a percentage of the value obtained from n = 1000 draws for each subject, taken to be the reference truth (dotted line at Y = 100%). a.u., arbitrary units; IQR, interquartile range; SD, standard deviation.

To provide context for interpreting basic descriptive statistics based on a limited number of observations, we framed the problem as one of comparing sub-sampled observations to an individual-level “ground truth” computed from longer duration observations from the same individual. We began with simulated distributions under two simplifying assumptions: known statistical distributions, and independence of observations within each simulated individual (no temporal relationships, i.e., the time series is stationary). We performed these simulations with four pre-defined distributions: two normal distributions (same mean, but different standard deviation (SD) values), and two skewed beta distributions with opposite skew directions (Fig 1A). Using these data generating functions, we simulated 5,000 individuals for each of the four distributions, with each simulated individual having 1,000 values (“days”) of data with arbitrary units (a.u.), from which a series of descriptive statistics were computed by sub-sampling from each simulated individual. The ground-truth values were defined by the result of using all 1,000 values per simulated individual for each derived summary metric. The results of sub-samples for n = 7, n = 14, and n = 30 nights are given for four summary metrics: mean, median, SD, and interquartile range (IQR), defined as the 25^th^ to the 75^th^ percentile value range (Fig 1B–1E). For each metric, the sub-sample value is normalized to the ground-truth calculation for each simulated individual, and reported as a percentage. Additional simulations using normal and log-normal distributions, ranging in sub-sample duration from 3–720 days, based on ground-truth of 1,000 values, are shown in S2 Fig in S1 File.

Several observations are notable from these simulations. For the two normal distributions, uncertainty in the mean and median are each clearly proportional to the SD (column 1 versus column 2), while no systematic directional bias is evident (the distribution is centered at 100% of ground truth). However, uncertainty in the measures of dispersion (SD and IQR) were the same for both of the two normal distributions. Furthermore, the SD and IQR both exhibited under-estimation bias for the smaller sub-samples, which was more prominent in the IQR calculations. Additional sub-sampling simulations and associated measures of dispersion from sub-samples of normal distributions with different SD values are given in S1 File.

For the skewed distributions, the mean and median exhibited distinct behavior depending on the direction of the tail. In the rightward tail simulations, the median contained markedly increased uncertainty (the error bar span was over twice as large for the median versus the mean), and also showed a directional bias toward over-estimation (whereas the mean showed no such bias). The SD and IQR both show an under-estimation bias, slightly more prominent in the IQR than in the SD. In the leftward tail simulations, the SD and IQR showed similar behaviors as the rightward tail simulations (including a more prominent under-estimation in IQR than in the SD). The mean and median showed much smaller ranges of uncertainty in the leftward tail case. This finding is attributed to the relative nature of the %-of-truth computation.

Comparing the normal distributions to the right skewed distribution, the relative uncertainty (error bars across the 5,000 simulations) was larger for the right skewed distribution regarding measures of centrality (Fig 1B, 1C). By contrast, uncertainty in the measures of dispersion were only modestly larger in the skewed compared to the normal distribution simulations (Fig 1D, 1E). For additional context, other measures of dispersion, besides SD and IQR, are shown for a normal distribution in S4 Fig in S1 File.

In these simulations, ground truth for the computed metrics of centrality and dispersion are from 1000 observations per individual. Prior work has attempted to answer similar questions, but using sub-sampling from short duration observations [7–10], such that the “truth” against which the sub-samples were compared was actually a short duration itself. We used simulations to compare results when “truth” is defined by only 7 or 14 nights of simulated data per individual. We hypothesized that short duration ground truth values would lead to less apparent uncertainty and thus faster apparent “convergence”, that is, overly optimistic inference about the suitability of short duration sampling. The results confirming this hypothesis are shown in S5 Fig in S1 File. For example, when 14 nights of total sleep time (TST) is taken as truth for each individual, sub-sampling shows a more rapid reduction in uncertainty of the mean TST estimate: the SD of the cohort is within approximately 5% of the “true” value (computed from 14 nights) by the 5th night sub-sample. In contrast, using the true mean value (7 hours), the SD of the sub-sampled values does not reach within 5% of truth until approximately 8 nights. A similar pattern is observed for computing the SD value from sub-sampling when the true SD is taken to be 14 simulated nights: there is less under-estimation bias, and more rapid reduction in uncertainty, compared to when the true SD is taken as the simulation parameter value (1 hour). Specifically, the under-estimation bias converges to within 5% of truth between nights 4 and 5, when 14 days is take as truth, whereas convergence to within 5% of the parameter value does not occur until night 7. Even more rapid apparent convergence is observed when only 7 nights of data is taken as the ground truth per individual. The uncertainty range (SD) for the computed SD is within 5% of truth at night 13 when 14 nights is taken as truth, whereas the simulated data shows such convergence of the SD requires approximately 180 nights.

### 3.2. Apple Heart and Movement Study: Longitudinal sleep duration and exercise data

The above simulations highlight uncertainties in basic descriptive metrics attributed solely to sub-sampling, since the distribution shapes are known and stationary. In real-world settings, neither the true distribution nor stationarity can be assumed. However, when longitudinal data is available, we have a unique opportunity to employ a similar empiric approach via within-participant sub-sampling, to answer pragmatic questions like, “if we only had 7 nights of data from each participant, how different might the measures of central tendency and dispersion be, compared to using much longer observation windows?” The AHMS cohort provides such an opportunity. The basic cohort characteristics are given in S1 Table in S1 File.

**Fig 2:**
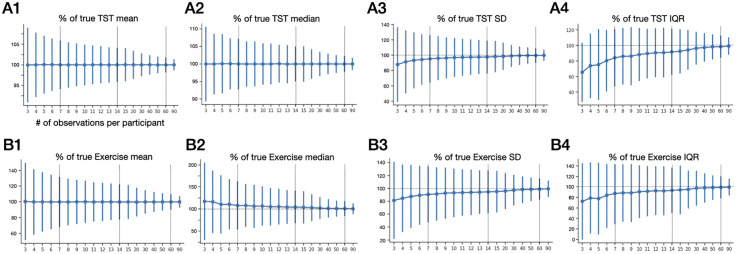
Descriptive metrics calculated across varying sample sizes of real world sleep and exercise data. In each panel, the mean (circles) and standard deviation (error bars; SD) of each summary metric is given for n = 40,441 participants, where the X axis indicates the number of observations drawn from each participant (X axis label in **A1** applies to all panels). The ground truth descriptive statistics for TST (hrs) and exercise (minutes) for each participant are determined from at least 90 values. Note the sample size on the X axis has non-linear increments after sample 15. The vertical dotted lines are for visual reference of samples 7, 14, and 60. IQR, interquartile range; SD, standard deviation; TST, total sleep time.

Fig 2 shows the results of random sub-sampling each individual’s sleep duration (total sleep time; TST) and exercise duration (minutes per day) values, where ground truth per participant is computed from at least 90 values for each metric (see Methods). The TST sub-sampling results show no directional bias for mean or median computations (Fig 2, A1 and A2). However, the exercise sub-sampling shows over-estimation bias for the median (Fig 2, B1 and B2). Although these results are reminiscent of the behavior of normal and right skewed distributions, respectively, we cannot infer the distribution shapes of empiric data based on this similarity (distribution testing is in section 3.4 below). The SD and IQR each exhibited under-estimation bias at lower sub-sample values, for both TST and exercise, before gradual convergence to within 5% of within-participant truth (Fig 2, A3, A4, B3, B4). Similar findings were evident if we restricted sleep sub-sampling to those with fewer than 90 nights (S6 Fig in S1 File). For comparison, sequential sub-sampling shows greater uncertainty and slower convergence as duration incrasses, compared to random sub-sampling (S7 Fig in S1 File). In summary, the empiric data sub-sampling quantified directional bias and convergence pace in real-world data, especially for the windows of observation of ≤14 nights that are typical of objective sleep tracking studies.

### 3.3. Apple Heart and Movement Study: Longitudinal sleep stages data

**Fig 3:**
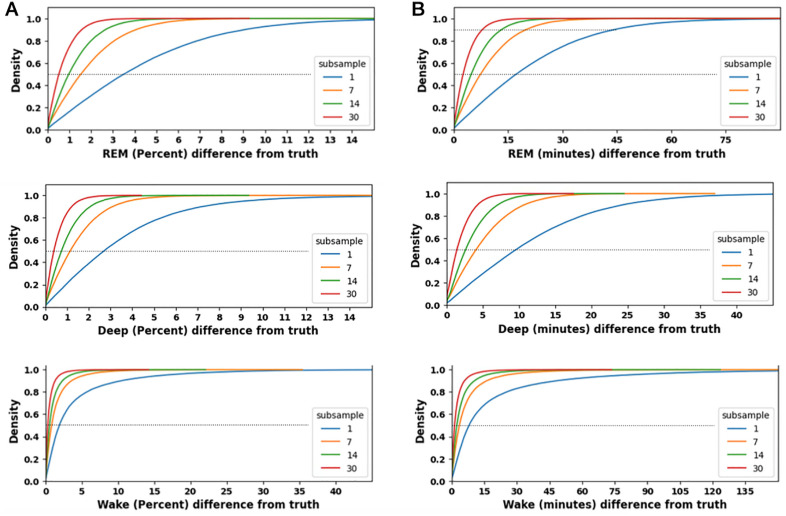
REM, Deep, and Wake amounts computed from sub-samples drawn from real-world sleep tracking. In each panel, CDF curves are plotted for sub-samples of size 1 (blue), 7 (orange), 14 (green), or 30 (red) nights, compared to the true amount within participant computed from at least 90 nights. The X axis indicates the deviation of the sub-sample from “truth”, for the percent (**A**) or the absolute amount (**B**) of REM sleep (first row), Deep sleep (second row), or wake after sleep onset (third row). The horizontal dotted line in each panel corresponds to the 50th percentile (density of 0.5); the intersection of each CDF with that line can be interpreted as half of the observations have at least that X-value of stage deviation from truth, or more. The deviations from truth are given as absolute values, independent of the direction.

Although sleep duration and its variability are common metrics used in epidemiology studies, some study designs use PSG testing to allow for stages within sleep to be assessed as another window into sleep physiology. PSG testing in large cohorts is even more limited in duration than actigraphy, with typically 1–3 nights of PSG per participant, as in the two largest meta-analyses of sleep staging data [1,2]. We sought to answer a similar question for sleep stages as we did for TST: “how different might an estimate of sleep stage duration (or % of TST) be, when using sub-samples of size 1, 7, 14, or 30 nights, as compared to estimates from longer duration observations?” Fig 3 shows this analysis from empiric sleep data, for stage duration and percentage. For each participant, the values computed from sub-samples are compared relative to a within-participant ground truth computed from longer observation windows. A sub-sample size of n = 1 night showed the most divergence, as expected, with median absolute difference of approximately 20 minutes for REM (and about 25% showing 30 + minutes of deviation). The median absolute divergence was approximately 10 minutes for Deep and Wake stages. The median divergence of REM as a percentage of TST was ~ 3.5%. For context, this divergence in REM sleep estimation is larger than the 3% difference in REM percentage reported in a cohort study associating REM sleep to dementia using a single night of PSG [18]. The observed divergence from “ground truth” reduces proportionately as the computations are made using 7, 14, or 30 nights to compute stage information within-participant.

### 3.4. Assessing normality and log-normality in simulated and real-world data

**Fig 4:**
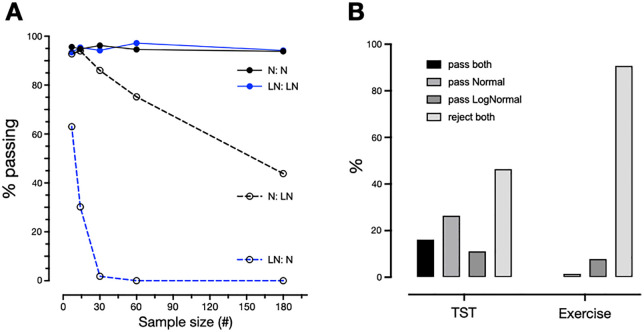
Tests of normality and log-normality, in simulated and empiric data. **A.** The percentage of a simulated cohort (n = 5000) passing Shapiro-Wilks tests of normality and log-normality for sub-samples of 7, 14, 30, 60, and 180 values. The 4 curves are the possible combinations of normal and log-normal distributions each being tested for normality and log-normality. The legend letters indicate the source distribution first, then the type of test applied. N: normal, LN: log-normal. **B.** The percentage of empiric data for sleep (left bars) and exercise (right bars) that passed Shapiro-Wilks tests of normality and/or log-normality. Exactly 180 values are included per participant in this analysis. TST, total sleep time.

In any setting of limited observations, the distribution shape may be challenging to ascertain. As a result, statistical decisions such as whether to the use a mean versus a median to summarize central tendency (and SD versus IQR for dispersion) may be unclear. To frame this challenge, we first simulated normal and log-normal (skewed) distributions (same shape as those shown in S1 Fig in S1 File). We then applied Shapiro-Wilk tests of normality and of log-normality to these simulated individuals (Fig 4A). At a sub-sample of 7 or 14 days, the data sampled from a normal distribution is similarly likely to pass either test. Even at 180 days, 43.8% of the simulations from the normal distribution still pass the log-normality test. True log-normal simulations are less likely to pass normality, even at 7 nights (although about two thirds still do pass at this small sub-sample), and this normality pass-rate rapidly approaches zero by 30 days.

Shapiro-Wilk tests were subsequently applied to the real-world observations of sleep duration and exercise minutes in AHMS participants. Each metric in each participant was tested for normality or log-normality with the two Shapiro-Wilk tests, using a fixed sample size for each participant (first 180 days each; Fig 4B). For both TST and exercise, the most common result was to reject both normality and log-normality, with this dual-rejection result being much more common in the exercise data (>90%); exercise passed log-normality in around 10% of participants. By contrast, the % passing only normality was 10x higher for TST than for exercise, while a considerable subset of TST passed both tests. These results suggest the heterogeneity of real-world sleep and exercise distributions exceed that predicted solely by sample size from the above sub-sampling simulations.

### 3.5. Short-sleep duration subsets: Simulations and real-world data

**Fig 5:**
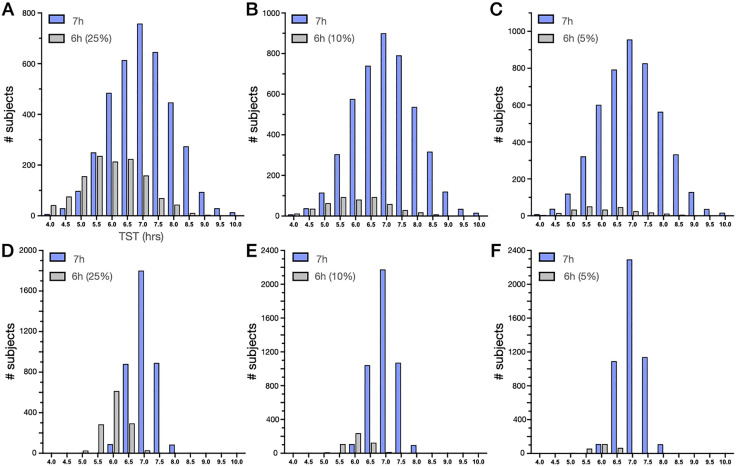
Relative distributions of TST values from a mixture of two simulated normal distributions. In each panel, the two distribution histograms are shown, in 30 minute bin widths, one for a simulated cohort with TST drawn from a normal distribution of mean and SD of 7 + /-1 hours (blue), and one for a simulated cohort drawn from a normal distribution of mean and SD of 6 + /-1 hours (gray). The X axis label (TST) in panel **A** applies to all panels. In the top row, the percentage of the total cohort (5,000) drawn from the 6-hour group is 25% (**A**; 1,250 subjects), 10% (**B**; 500 subjects), or 5% (**C**; 250 subjects. Each subject contributes a single TST value drawn from its generator function. In the bottom row, the same cohorts are used, but each subject contributes their mean of 7 nights. See Supplemental Figure S4 Fig in S1 File for visualization of the mixtures in panels **A**-**C** blind to cohort identity. TST, total sleep time.

A single night of objective sleep recording is sometimes used in epidemiology studies of sleep duration. One such circumstance involves quantifying “short sleep”, as has been done with PSG testing in the context of subjective versus objective insomnia phenotypes [19]. In this setting of a single objective TST value, duration-based labels such as “short sleep” (e.g., less than 6 hours), may be viewed as an extreme form of under-sampling. Again, we approach this question first with simulations, and then with real-world objective sleep tracking data from AHMS participants. For the simulations, we created a mixture of two sub-cohorts, one drawn from a normal distribution with mean and SD of 7 + /-1 hours, and a “short sleep” cohort drawn from a mean and SD of 6 + /-1 hours. Fig 5 illustrates the distribution histograms of TST values for each sub-cohort, across three prevalence values for the 6-hour group: 25%, 10% or 5% of the total cohort (the remaining fraction being drawn from the 7 + /-1 hour distribution; Fig 5A–5C). We can visualize and estimate the relative likelihood of any given simulated individual belonging to the 6-hour or 7-hour cohort if we assign their bin using only a single TST value. For example, consider the bin centered at 6 hours of TST: when the total cohort mixture includes 25% of the true 6-hour individuals, an observation of 6 hours is about twice as likely to come from the true 7-hour sub-cohort than from the true 6-hour sub-cohort. When the 6-hour sub-cohort represents a smaller subset, say, 10% or 5% of the total, a single observation in the 6-hour bin is 7x or 20x, respectively, more likely to have come from random variation of a true 7-hour sub-cohort individual. In other words, under these simplified but plausible assumptions, the vast majority of single-night 6-hour TST bin observations actually come from random variation of the 7-hour group, and thus are mis-classified as “short” sleepers. Even observations of 4 hours will be falsely considered “short” (i.e., they come from variation of the 7 hour sleepers), if the prevalence of the short sleep group is 5%.

To assess the benefit of using more than one observation to compute an individual’s TST for the purposes of assigning a short sleeper phenotypic label, we repeated the simulations where each individual’s TST value is computed from the mean of 7 simulated nights (Fig 5D–5F). The distribution histograms are now more centered around the simulated mean values, as expected from this increased number of observations per individual. When the 6-hour sub-cohort is 25% of the total, this 7-night averaging shows that observations in the 6-hour bin are now 7x more likely to have come from the true 6-hour sub-cohort, versus from the 7-hour sub-cohort (Fig 5D). However, at 10% prevalence of the 6-hour sub-cohort, an observation in the 6-hour bin is only about twice as likely to have come from the 6-hour as the 7-hour sub-cohort (Fig 5E), and at 5% prevalence (Fig 5F), a 6-hour observation is about equally likely to come from either sub-cohort.

In summary, although repeated observations markedly improve the accuracy of a given bin assignment in this simulation, if the short sleep group is a small portion of the total cohort, observations distant from the true mean (such as “short sleep”) may be more likely to have arisen from random variations of the dominant group (in this simulation, the 7-hour group), than from a low-prevalence group with a truly shorter mean TST value. Of note, it can be challenging to visualize in population level histograms that contain mixtures of sub-groups, which can visually resemble single normal distributions even when generated from mixtures of two normal distributions (S7 Fig in S1 File).

**Fig 6:**
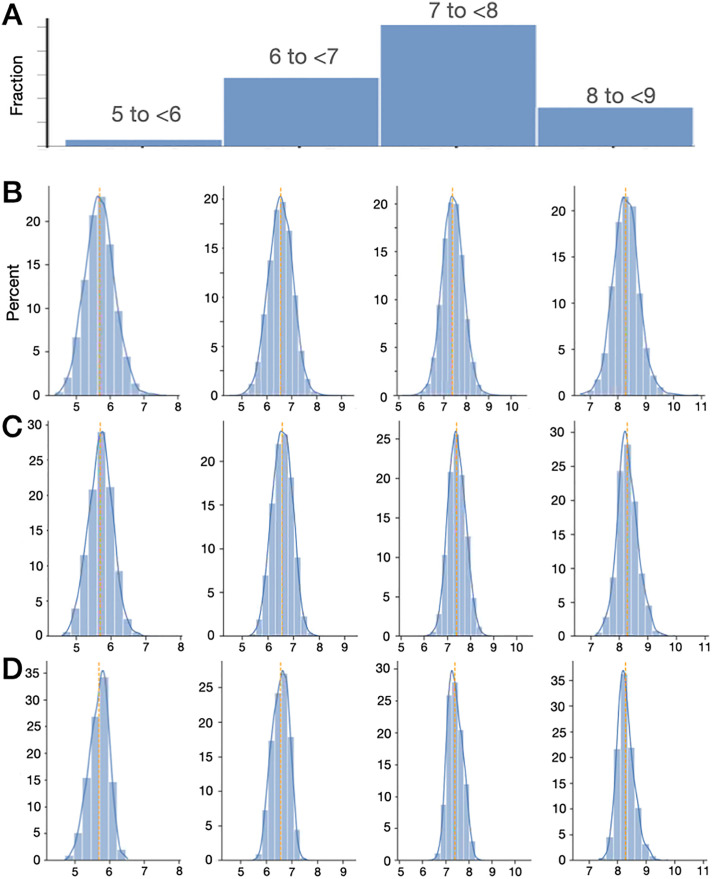
Distributions of TST values from real-world data at varying sub-sampled observation windows. **A**: Ground truth TST binning of the real-world cohort, using 90 + nights of TST to calculate each participant’s mean TST. Due to small samples in extreme bins, the < 5 and >9 bins are not shown. In panels **B**-**D**, histogram distributions are shown for TST durations, one value per participant, calculated from sub-samples of 1 **(B)**, 7 **(C)**, and 14 (**D**) observations each. Each of the four columns correspond to participants classified in each of the 1-hour bins in panel **A**, to illustrate the spread of possible observations among individuals assigned ground truth bins of 5-6, 6-7, or 7-8 hours (columns) using 90 + TST values, when a random sub-sample is taken (rows). The orange vertical dotted line is the median value. The X axes in **B**-**D** are TST values (hours), and the Y axes are % in panels **B**-**D**. TST, total sleep time.

Real-world TST observations allow exploration of this phenomenon more directly, again taking advantage of longitudinal data defining the “truth” for each participant. We computed the mean TST using at least 90 nights of sleep duration data per participant, which is taken as ground truth, against which the random sub-samples are compared. Fig 6A shows the distribution of TST values using this approach, in 1 hour bins. We drew random sub-samples of 1, 7 or 14 nights (Fig 6, B-D respectively) from participants in each of 4 “true” bins of 5 to <6 hours, 6 to <7 hours, 7 to <8 hours, and 9+ hours. The results illustrate how intra-individual variation can lead to bin mis-assignment (bins filling outside of the “truth” category), which is mitigated by increasing the duration of observations (narrower distribution of values, fewer of which land outside of the true bin).

## 4. Discussion

This study utilized a combination of simulations and real-world longitudinal data to provide an inferential framework around the limitations of short duration sampling of temporally variable data types such as sleep duration. We found that directional biases and uncertainty ranges depend on three main factors: a) the sample size of observations within each participant, b) the descriptive statistic used to capture centrality or dispersion, and c) the distribution shape of the data (normal or skewed).

### 4.1. Observational studies using objective sleep metrics

The literature commonly utilizes one or a few nights of PSG data, or 1–2 weeks of actigraphy or diary data, to draw inferences across a range of research questions. Normative data regarding sleep metrics such as TST and sleep staging was published in two meta-analyses, which drew mainly from studies of 1–3 nights of PSG data. Fig 3 provides a quantiative estimate of uncertainty in the mean values of sleep stage metricsc computed from short durations, while the mean values are not expected to exhibit directional bias. Similarly, research studies regarding insomnia with objective short sleep, an important advancement in insomnia phenotyping, used single night PSG data per assessment, such as the Penn State cohort [19], as well as the Sleep Heart Health Study cohort [20]. The simulations of Figs 5 and 6 suggest likely label mixing in such studies, which may result in under-estimation of the risks associated with short sleep. Observational cohort studies often use short duration actigraphy such as the UK Biobank [21], the Multi-Ethnic Study of Atherosclerosis [22], and the Jackson Heart Sleep Study [23]. These cohorts have resulted in numerous publications regarding the health associations of sleep duration and sleep variability [24–27] (although other studies failed to show health outcome correlates with sleep, such as the HypnoLaus cohort [28]). The current work suggests that risks associated with nightly variability might be under-estimated when that exposure is computed from short durations. Until such efforts are replicated with longer duration tracking, it remains unclear how the inferential conclusions might differ, since not all tested hypotheses can be assumed to bias toward the null given uncertainties from sub-sampling (see section 4.2).

Key limitations when linking sleep metrics to adverse health outcomes include assessment method (e.g., PSG versus actigraphy versus consumer trackers) and the relative impact of sub-sampled observation windows both from a statistical standpoint (as in the current work) and with respect to representativeness of the sampling window (as in the issue of non-stationarity). In the current work, we reduced the impact of non-stationarity in real-world sleep tracking data by random sub-sampling within each participant, in service of bounding the statistical concerns associated with small observation windows. However, in realistic tracking scenarios, non-stationarity is likely quite common. Indeed, sub-sampling consecutive nights shows even more pronounced under-estimation bias in SD estimates, including a slower pace of converging to the within-individual true value derived from longer observations (S6 Fig in S1 File).

Considering nightly variability in sleep metrics is crucial for study planning and interpretation, but the answer may be influenced by many intrinsic and extrinsic factors. For example, the night-to-night variability of any sleep metric may be a complex combination of behaviors potentially impacting sleep [29–31] and individual vulnerability to such perturbations. Such factors would compound any inherent (biological) stochasticity [32] that may be observed even under constant behavioral conditions. External pressures spanning work, social, and family contexts add further variability in naturalistic settings. The combination of these factors may differ among individuals, and may differ over time within individuals, the latter contributing to non-stationarity and thus questions of representativeness for any given observation window. How long to observe an individual to characterize non-stationarity is beyond the scope of the current work, but is predicted to depend on the cycle characteristiccs (if cyclic), and on the magnitude and temporal pattern (if isolated or intermittent rather than cyclic). Even a theoretically cyclic non-stationarity like weekend versus weekday behavior might not manifest equally on every weekend.

Ultimately, the practical dilemma is that representativeness of an observation window for any analytic purpose is unclear in naturalistic data, without having longer-term data against which to compare (in which case, shorter windows might not be utilized except perhaps to evaluate hypotheses of non-stationary factors). The current work is solely addressing issues with routine statistics of centrality or dispersion, while more complex patterns and non-stationarities are predicted to be even more challenging to surmise from short duration samples. Longitudinal tracking overcomes some of the limitations associated with relatively short observation windows, but inter-individual variability needs to be recognized even when observations are relatively long.

### 4.2. Studies of intra-individual variability

Bei, et al. summarized numerous studies that tracked sleep with actigraphy, most of which had durations of 7–14 nights [3]. Actigraphy has well known limitations in sleep-wake classification, with typically high sensitivity for sleep but low specificity [33], such that TST is over-estimated in proportion to the amount of true wake, that is, as sleep efficiency decreases. Even if actigraphy were as accurate as PSG, our current results illustrate statistical and conceptual limitations apply to the interpretation of intra-individual variability (IIV) obtained from relatively short observation windows. In their meta-analysis, Bei, et al. reported that the SD of sleep duration was the chosen metric used to describe sleep variability in the majority of studies.

Within-individual sleep variability has been linked to a variety of adverse health outcomes, such as mental health [34], cardiovascular health [24], metabolic health [35], and obesity [36]. Both the uncertainty of the estimate, and the under-estimation bias of the SD, are predicted to bias hypothesis linking SD to negative health outcomes toward the null in epidemiological studies. In other words, potential health associations with metrics of sleep variability may not reach significance, or if they do, the magnitude of association may be under-estimated. As one empiric example of this issue in the current study, we computed the Spearman correlation coefficient between age and the SD of TST, and found that it progressively decreased from a value of −0.22 using >=90 values per participant, to smaller values of −0.17, −0.13, or −0.08, using progressively shorter observation windows of 30, 7, or 3 nights per participant, respectively.

On the other hand, uncertainty and underestimation of the true SD could also theoretically bias certain analyses toward an inflated effect, in the context of confounder analyses. If sleep variability is considered a confounder, then uncertainty and/or under-estimation of SD predicts that the influence of SD might be incompletely accounted for in a model, which could bias toward over-estimation of risk. If sleep variability is a risk factor of interest, opposing biases can arise in the “causal” link (say, TST SD being a risk factor), and the control variable link. Consider a cohort study aiming to associate sleep variability with hypertension, after controlling for age. Insufficient sleep tracking duration could result in the true relation of age to sleep variability being under-estimated, leading to incomplete control of age as a confounder), while the link of TST SD to hypertension might be under-estimated.

The specter of non-stationarity is likely in naturalistic sleep data, given the myriad of factors known to impact sleep on any given night, and the fact that those influences themselves may vary over time. Consider the seemingly simple issue of weekend versus weekday sleep behavior differences, which could impact descriptive statistics computed from a short sampling window. A single week of TST observations divided into weekday and weekend subsets represent even further sub-sampling risk (from 7 nights, to two and five night samples, respectively). Such weekend effects can markedly impact the SD calculation even when longer windows are available. S8 Fig in S1 File shows with simulations how a longer weekend TST impacts the TST SD, whether the weekend effect repeats or occures in isolation. Many other common factors contribute to sleep patterns deviating from simplifying assumptions, such as work schedules, monthly cycles, seasons, and numerous other episodic transients (pain, mood, stress, life events, family obligations). Influences on sleep that have long effect lag-times, or that themselves change slowly, could require months or longer to assess, such as seasons, changes in weight, or adoption of a new habit that takes time to master. These and other transient perturbations (e.g., illness) may also be analyzed through the lens of change-point analysis. Larger observation windows increase confidence in assessing distribution shape, and reduce the biases described herein regarding short observation windows. They also allow more complex analysis of stationarity or change-point approaches to identify periodic (e.g., weekend or monthly) or non-periodic fluctuations in the data (e.g., transient illness).

### 4.3. Matching statistical assumptions to phenotypes of interest

It is commonly understood that confidence in any computed statistic is proportional to sample size. However, in short duration studies of sleep, confidence bounds and error bars are more likely focusing on the population sample size, rather than on the computed sleep metrics per participant (mean, SD, etc). Explicit accounting for this latter issue is important, since it can bias hypotheses toward or away from the null, depending on the circumstances. One option to mitigate is to assess comparative goodness of fit metrics to choose the more favorable fit. While metrics such as the median and IQR may be chosen as a conservative approach not involving a normality assumption, it is worth noting that the IQR shows more prominent under-estimation than the SD, even for a true normal distribution (Fig 1). Understanding distribution shape also has implications for handling outliers. Absent a strong prior belief as to the true expected distribution, it would be difficult to interpret an apparent outlier point in a small sample, which could easily be part of a tail of a skewed distribution that was simply too sparsely sampled to properly visualize the tail.

Variability might itself be part of a phenotypic axis (S9 Fig in S1 File), for example the TST SD is inversely correlated with age, while female participants had a slightly higher SD value than males (median of 1.04 hours versus 0.98 hours; while statistically significant due to the large sample size, the absolute difference was only 3.6 minutes). Ultimately, the question of how long to track sleep depends on the goals of analysis as well as the statistical characteristics of the sleep measurements and potential sleep influencers under consideration. For the common circumstance of existing datasets, the cohort sample size and the duration of tracking per individual is already set. For such cases, this study provides a framing to interpret howeer many nights are available, so that limitations can be more concretely described, if not accounted for. For the planning of future studies, there may be a resource and/or participant burden tension between sample size and duration of tracking. S9 Fig in S1 File uses simulations to frame this tension in a statistical sense: if under-estimation bias of the SD computation is important for a study and there is flexibility in protocol design, the simulations suggest that the path to mitigation lies in duration of tracking per participant, more so than boosting cohort sample size. If by contrast participant burden is a prime concern, boosting sample size improves confidence in population metrics, in addition to facilitating sub-group explorations.

### 4.4. Limitations

The current analysis is based on a subset of the AHMS cohort, and the extent to which the results about distribution shape and heterogeneity generalize to other populations, or using other tracking technologies, is unknown. However, the measures of variability of TST obtained in this cohort, as measured by Apple Watch first party sleep tracking, are similar to the range reported in other studies (e.g., SD of TST in the range of 1–1.5 hours). The relevance of statistical principles associated with relative-under-sampling are demonstrated via simulations, meaning, the uncertainties and biases associated with small samples can occur even under perfect measurement information assumptions. We did not address the potential for non-stationarity of sleep or exercise habits over time. Non-stationarity is likely common, and is predicted to further complicate measures of centrality and dispersion, as we observed in simulations and empiric sleep data.

## Supporting information

S1 File 10 Figures and 1 Table. (PDF)
