# Supplementary material for: Naturalistic sleep tracking in a longitudinal cohort: Uncertainty and bias in short duration sampling
Source: PLoS One. 2025 Nov 3;20(11):e0334950. doi: 10.1371/journal.pone.0334950 (PMC12582441; doi:10.1371/journal.pone.0334950)
Supplement: S1 File — (PDF) [file pone.0334950.s001.pdf]

Metric	Value
Age (years)	46.3 (13.6)
Sex	71.5% M, 28.3% F
BMI (kg/m^2)	28.2 (6.0)
Mean TST (minutes)	412 (43)
SD TST (minutes)	61 (15)
Mean Exercise (minutes)	37.7 (25.6)
SD Exercise (minutes)	26.4 (13.7

S1 Table. Cohort characteristics
Values are mean (standard deviation, SD, in parentheses) for each continuous variable, and percent for biological sex. Sleep and exercise metrics are first computed as a mean value per participant, and then the population mean and SD are provided in the table. BMI, body mass index; kg, kilograms; m, meters; SD, standard deviation; TST, total sleep time.

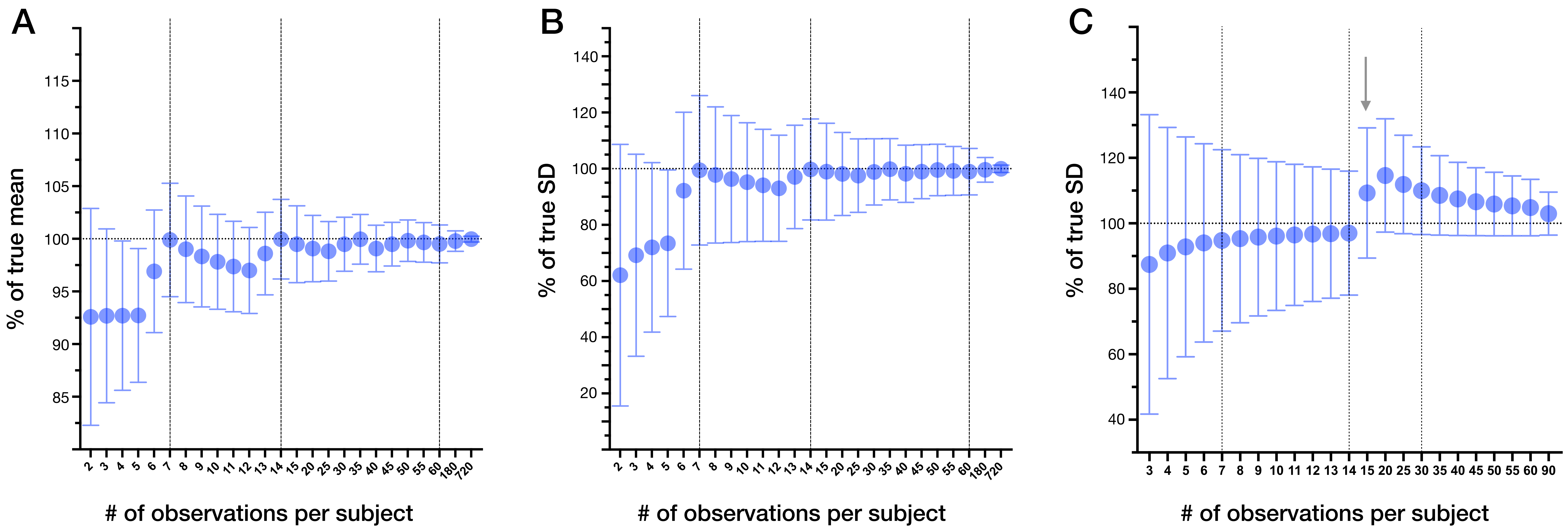

S1 Fig. Mean and SD of sub-samples computed from a simulated cohort with longer weekend sleep.
In this simulation, weekdays are drawn from a normal distribution of 7±1 hours of sleep duration, and weekends are drawn from a normal distribution of 8±1 hours. In each panel, the mean (circles) and standard deviation (error bars; SD) of each summary metric is given for n=5000 simulated subjects. The X axes in panels A and B indicate the number of sequential (not random) observations drawn from each simulated subject, beginning on a Monday such that the weekend days are numbers 6 and 7 (when jumps are seen from the longer weekends), then dip again as more weekdays accumulate, until eventually there is convergence as more weekends are incorporated. Note the sub-sample sizes (X axes) have non-linear increments after day 15. The vertical dotted lines are for visual convenience at 7, 14, and 60 day windows. The computed mean and SD values for each sub-sample are normalized to the value obtained from n=1000 draws, taken to be the reference truth for each simulated subject, such that the Y axes represent the % of this true value. Panel C is arranged similarly, but with the first 14 days from a distribution of 7±1 hours of sleep, then a single weekend drawn from a distribution of 9±1 hours of sleep (arrow), then back to 7±1 hours through day 90. SD, standard deviation.

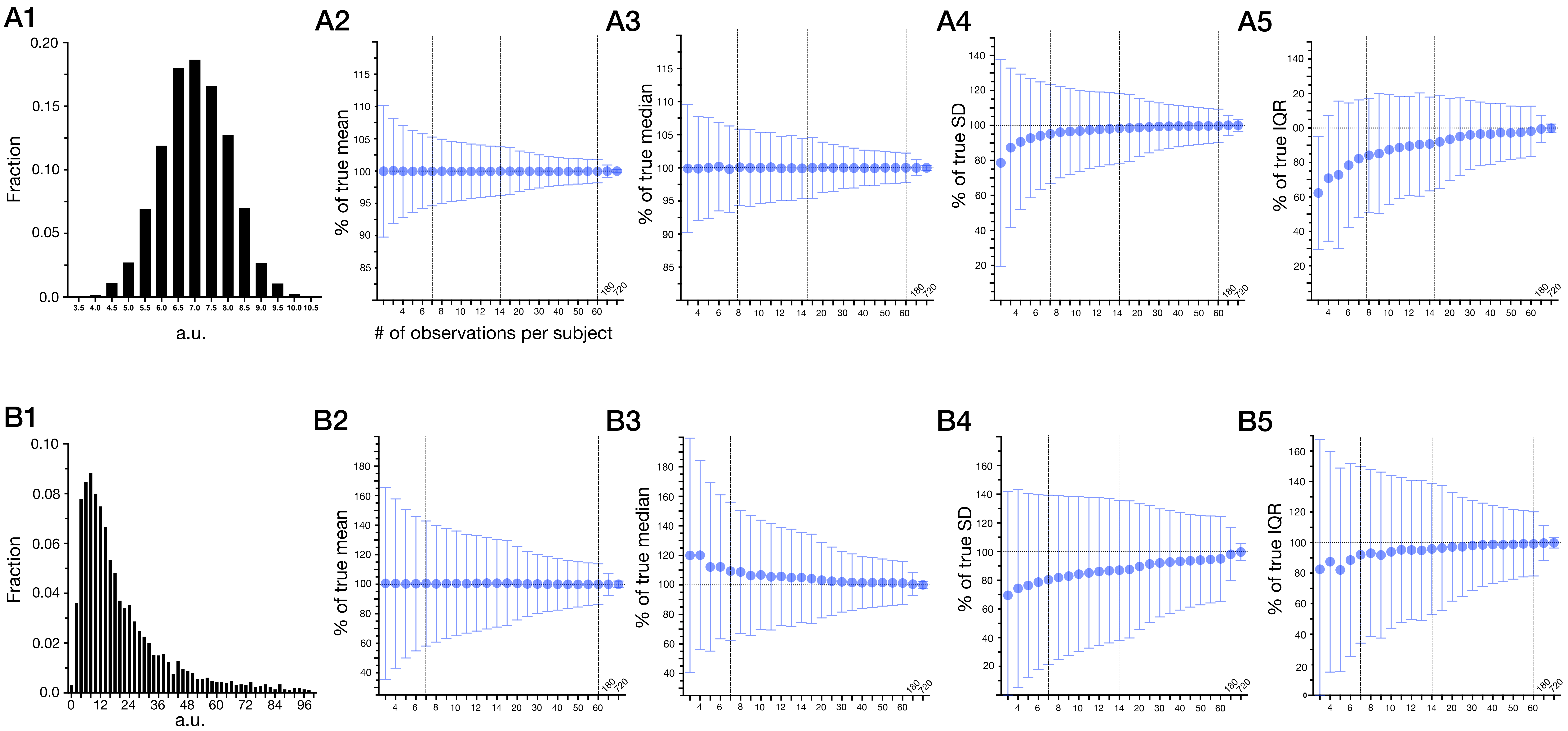

S2 Fig. Descriptive analyses across varying sub-samples drawn from a simulated normal or log-normal distribution.
Panel A1 and B1 show the distribution of 5000 random values from a normal or log-normal distribution, respectively (parameters of 7+/-1, and 2.7(0.9). In the remaining panels, the mean (circles) and standard deviation (SD; error bars) of each summary metric is given for n=5000 simulated subjects, where the X axis indicates the number of observations drawn from each simulated subject (X label in A2 applies to all panels). Note the sub-sample sizes (X axes) have non-linear increments. The vertical dotted lines are for visual convenience at 7, 14, and 60 sample windows. The summary metrics are normalized to the value obtained from n=1000 draws, taken to be the reference truth for each simulated subject. a.u., arbitrary units; IQR, interquartile range; SD, standard deviation.

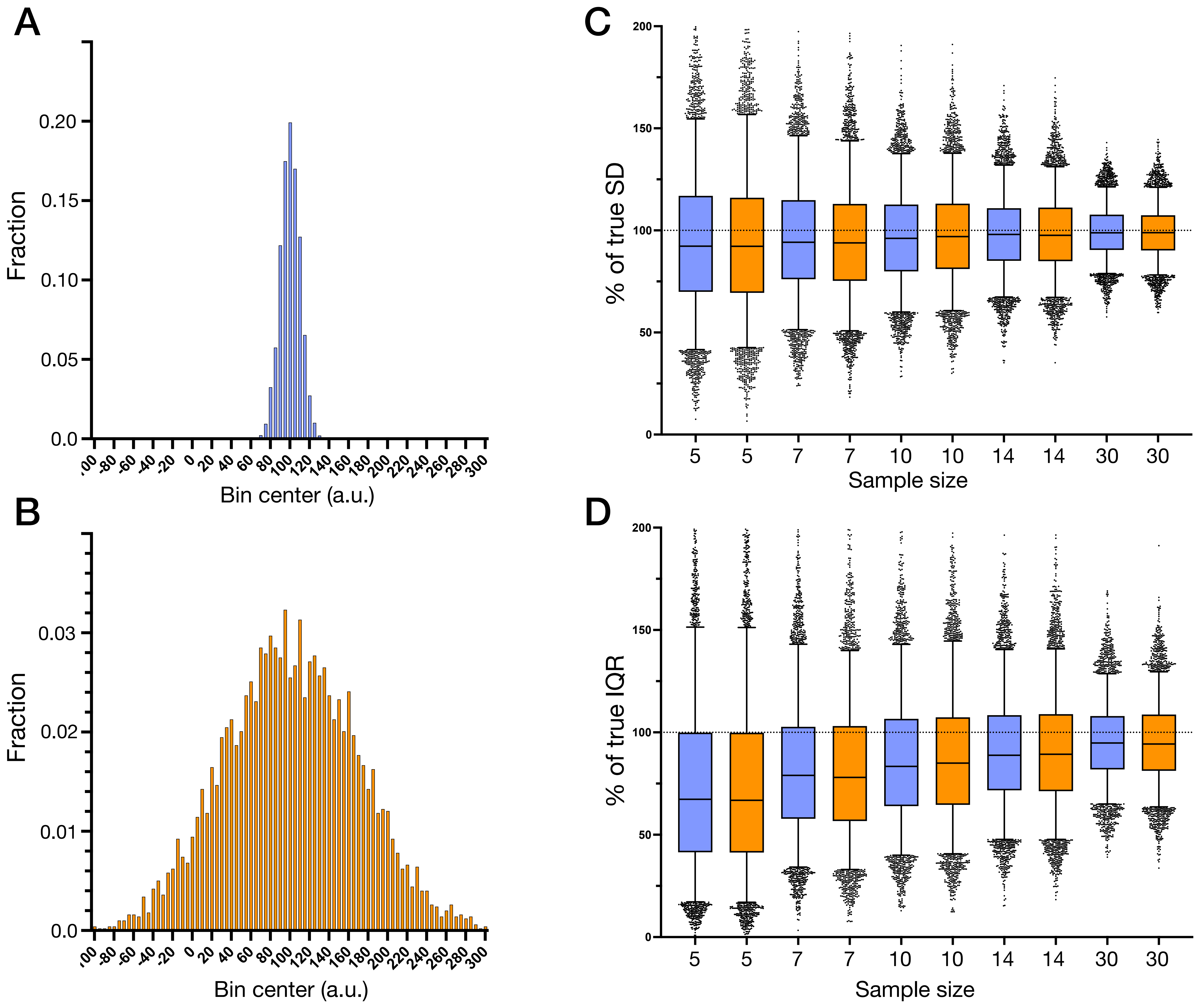

S3 Fig. Measures of dispersion in sub-samples drawn from normal distributions with small versus large standard deviation values.

A. Histogram of 5000 values drawn from a normal distribution with mean 100 and SD 10 (a.u.).
B. Histogram of 5000 values drawn from a normal distribution with mean 100 and SD 70 (a.u.).
C. Distribution of SD values computed from sub-samples of the narrow (blue) versus wide (orange) distributions corresponding to panels A and B, respectively. The box plots show the median, IQR, 5-95%ile, and dots for individual points beyond the whiskers. The values are scaled as a percentage of the "true" value computed from 1000 values per simulated individual (horizontal dotted line).
D. Distribution of IQR values computed from sub-samples, as in panel C.
a.u., arbitrary units; SD, standard deviation; IQR, interquartile range

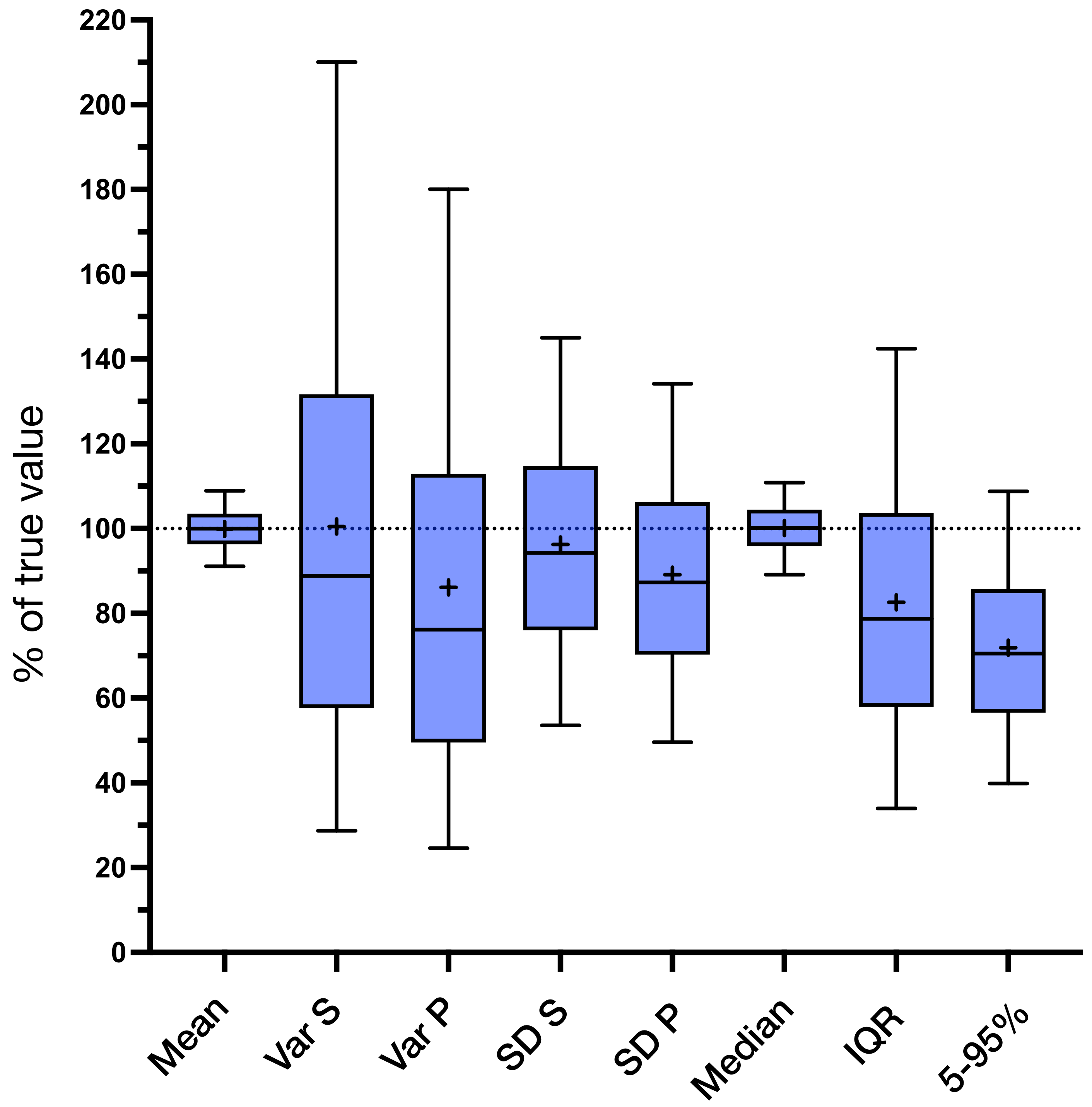

S4 Fig. Measures of centrality and dispersion, for sub-samples of n=7 nights, drawn from a simulated normal distribution.
In this simulation, values are drawn from a normal distribution of 7±1 hours of sleep, for 1000 nights each of n=5000 simulated individuals. The distributions across these 5000 simulated individuals are shown as box plots for centrality (mean and median) and dispersion (variance, standard deviation, interquartile range (IQR), and 5th to 95th percentile range), in each case normalized to the true values defined by using all 1000 nights per individual. For variance (Var) and standard deviation (SD), two versions are shown for each, the population (P) and sample (S) versions of the computations (Excel command versions), which use a denominator of either n or n-1, respectively. Each box plot shows the median, IQR, and 5-95%ile (whiskers), as well as the mean ("+"), for the values obtained from 5000 simulated subjects.

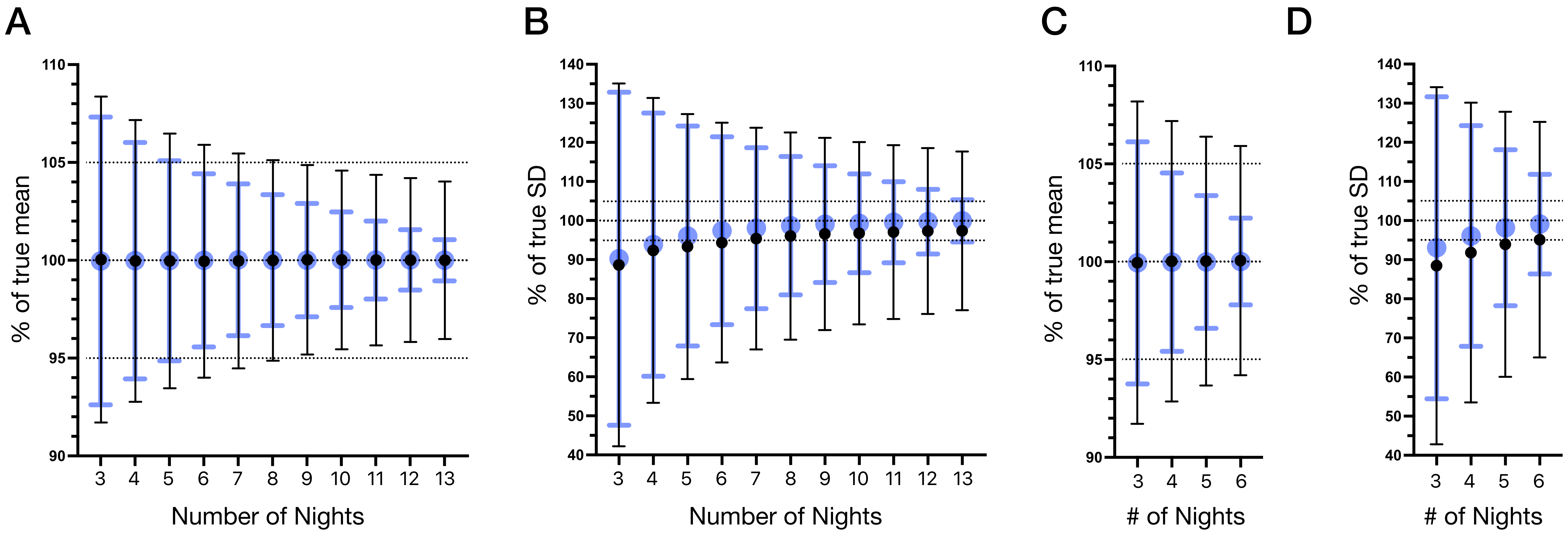

S5 Fig. Sub-sampling from a simulated cohort using short samples as truth.
In this simulation, TST values are drawn from a normal distribution of 7$\pm$1 hours. In each panel, the "truth" is defined either by the simulation parameter (black circles and SD error bars), or from a small duration sample (blue circles and SD error bars) of only 14 nights (panels A and B) or 7 nights (panels C and D). In each panel, horizontal dotted lines are for visual convenience at Y axis values of 95%, 100%, and 105% of the truth, so that the relative "convergence" toward 100% as sub-sample size increases can be visualized more easily. For each of the 5,000 simulated individuals, the computed mean and SD values of TST are normalized to either the truth from the simulation parameter (7 hrs TST, SD of 1 hour), or normalized to the value obtained for either 7 or 14 nights of TST, such that the Y axes represent the % of this true value. SD, standard deviation.

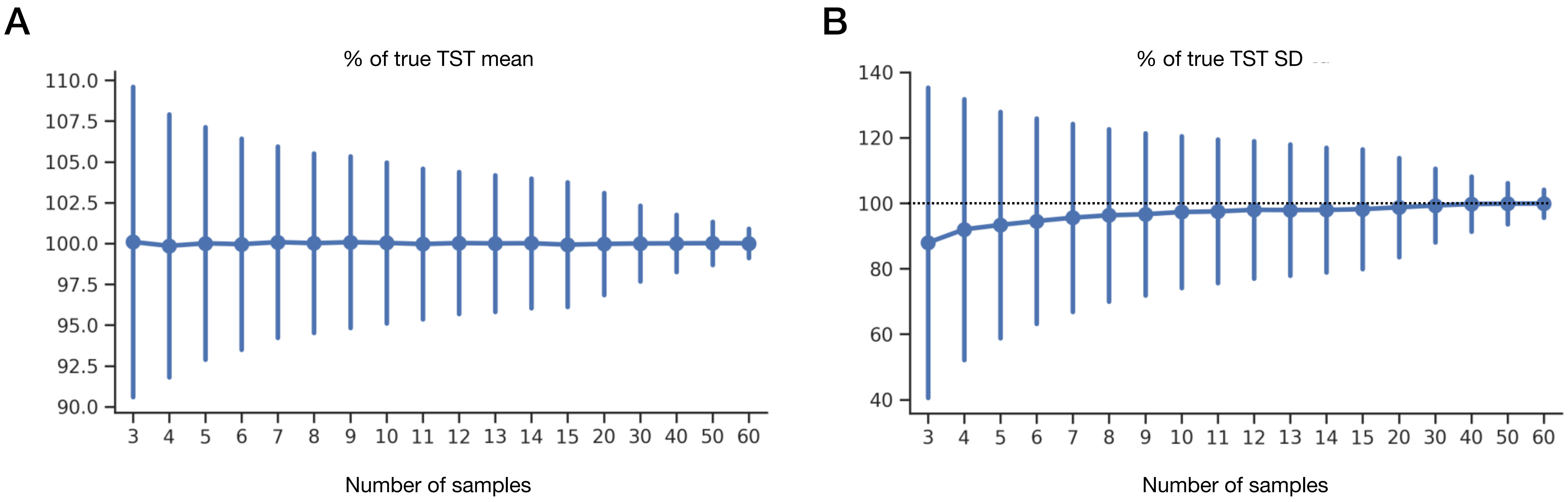

S6 Fig. Mean and SD of TST calculated across varying sample sizes from individuals with 60-89 TST values.
In each panel, the mean (circles) and standard deviation (error bars; SD) of each summary metric is given for n=9.282 participants who had more sparse data (fewer nights of sleep data) than the main cohort where the requirement was >=90 nights). The X axis indicates the number of observations drawn from each participant (X label in A1 applies to all panels). The ground truth descriptive statistics for TST (hrs) and exercise (minutes) for each participant are determined from at least 60 values. Note the sample size on the X axis has larger increments after sample 15. A horizontal dotted line is shown at 100% on the Y axis in panel B for visual reference. SD, standard deviation; TST, total sleep time.

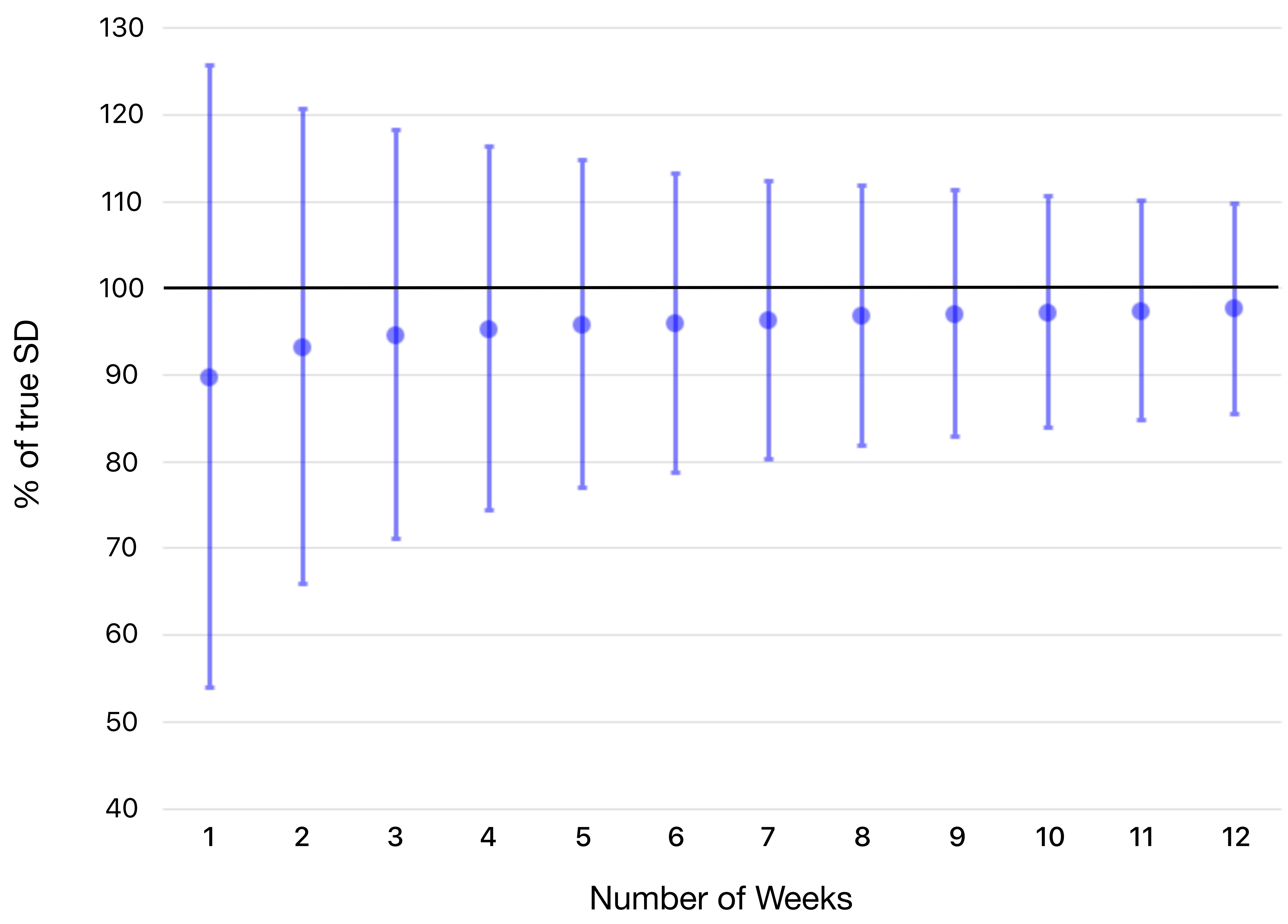

S7 Fig. Sub-sampling consecutive nights from empiric sleep data.
This analysis includes participants with at least 85 nights of TST data in a 90-day window (mean of 88 nights each). The strict data presence requirement was implemented to minimize the impact of missing data for computing metrics using sequential calendar nights of data. Ground truth for the SD was taken from all available data per participant (mean of 313 nights each). The SD was computed from consecutive sub-samples in 1 week increments, such that the X axis ranges from 7 nights to 84 nights. The 90 day period of observation began for all participants on the same date, which was a Sunday night. The Y axis is the % of true SD, which is computed for each individual. The average SD in this group was 59 minutes. The mean (circle) and SD (blue error bars) are computed from n=5,790 participants included in this analysis. SD, standard deviation.

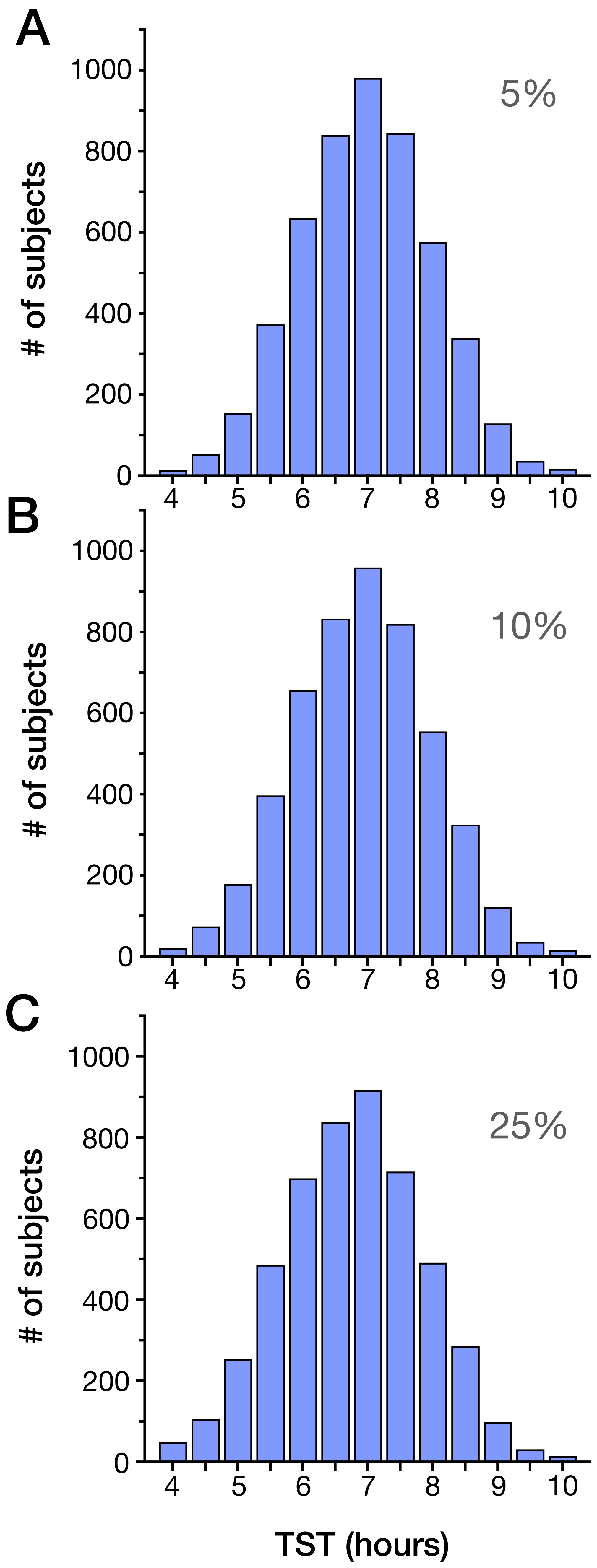

S8 Fig. Distributions of simulated cohorts with a mixture of two normal distributions. In each panel, the binned histogram distribution is shown for simulated cohorts of n=5,000 individuals, with each one contributing a TST value drawn from a normal distribution of either 7+/-1 hours or 6+/-1 hours. The relative contribution of the shorter (6hr) TST sub-cohort is either 5% (A), 10% (B), or 25% (C). Note that these data are the same as shown in Figure 5 of the main manuscript, which shows the corresponding sub-cohorts broken out in interleaved bar graphs). TST, total sleep time.

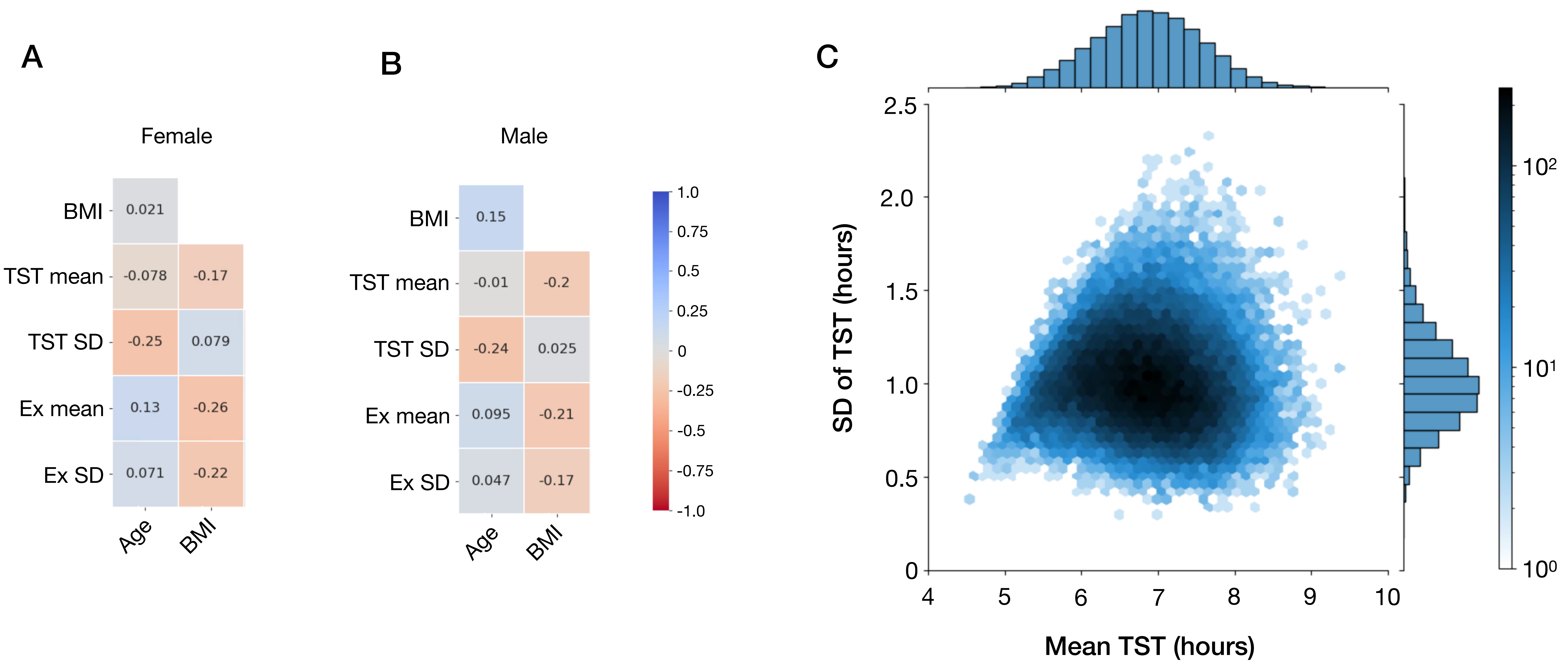

S9 Fig. Potential phenotypic correlates.
Panels A and B contain Spearman correlation values for the indicated demographic and HealthKit data, separately computed for female and male participants. The values are given for descriptive purposes only; the large sample size is overpowered to find even very small R values statistically significant. The color legend represents Spearman R values, from 1 to -1 range, and applies to both panels. Panel C shows the SD of TST, computed per participant, on the Y axis, against the mean TST, also computed per participant, on the X axis. The marginal distributions are given in corresponding histograms for each of these two metrics. The color in each hexagon cell corresponds to the legend shading scale for the number of participants in each hexagon. There was no apparent linear relationship, with a linear regression yielded a slope value of 0.00. SD, standard deviation; TST, total sleep time. Ex, exercise; BMI, body mass index; SD, standard deviation; TST, total sleep time.

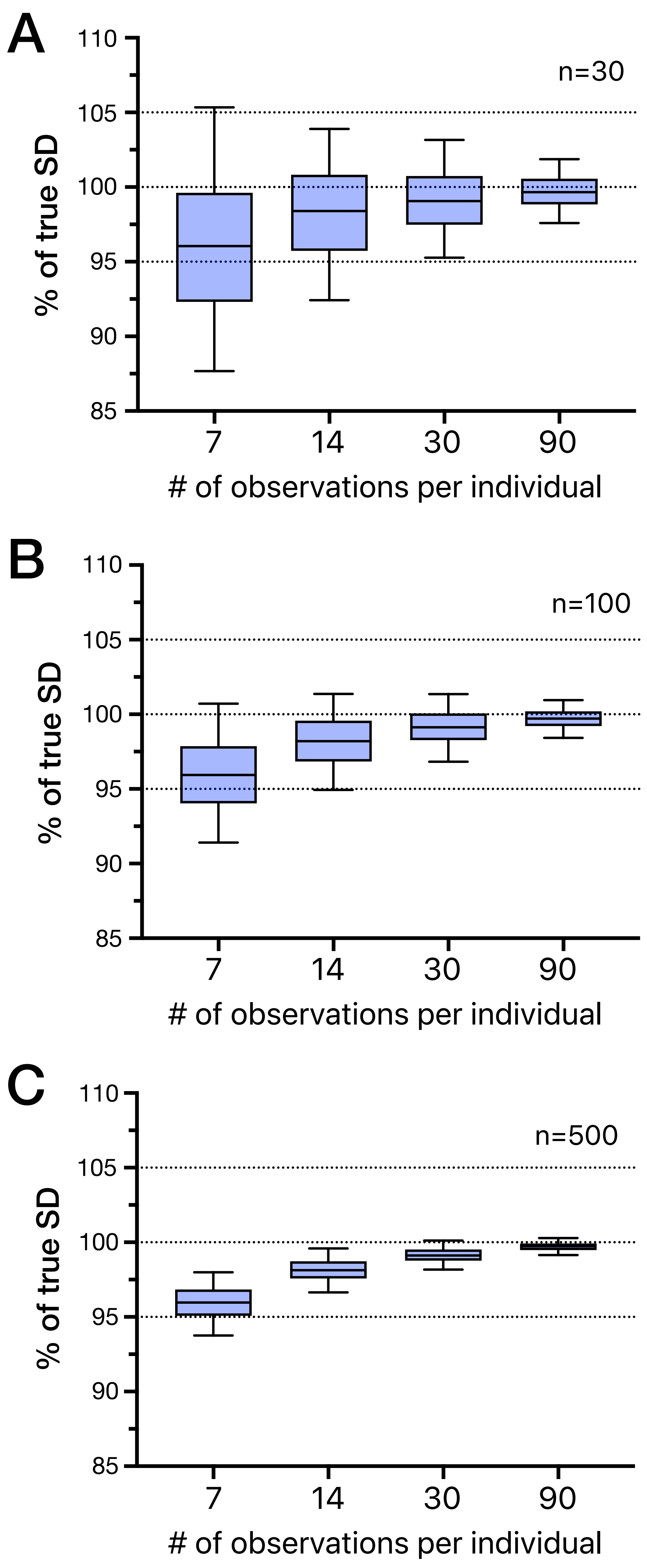

S10 Fig. Standard deviation estimates across simulated combinations of sample size and observations per individual.
Simulations were performed with random values from a normal distribution (arbitrary units, mean of 100, standard deviation (SD) of 30). The number of individuals in each simulated cohort was n=30 (panel A), n=100 (panel B), or n=500 (panel C). The number of observations per individual, from which the SD was computed, was 7, 14, 30, or 90 (x-axis in each panel). 1000 simulated experiments were run for each pair of cohort sample size and observations per individual. The distribution of SD values from these experiments is given in the box plots, where the whiskers are the 5-95% range. In each experiment, the SD obtained was normalized as a percentage of the true value (y axis in each panel). Note that the distributions (box plots) in this simulation are of the cohort level estimates of SD across simulated cohorts (and thus are smaller than the distributions shown in the main text, which are summarizing individual level estimates from a simulated cohort size of 5,000). SD, standard deviation.
